# Gender Parity in Authorship of Published Randomized Clinical Trials in Stroke Neurology From 2000 to 2021

**DOI:** 10.1001/jamanetworkopen.2022.2423

**Published:** 2022-03-15

**Authors:** Noor F. Shaik, Ali A. Saherwala, Diana L. Tzeng

**Affiliations:** 1 Department of Neurology, Sidney Kimmel Medical College, Thomas Jefferson University, Thomas Jefferson University Hospital, Philadelphia, Pennsylvania

## Abstract

This cross-sectional study examines proportions of male and female first and last authors in randomized clinical trials on stroke neurology published from 2000 to 2021.

## Introduction

Gender parity is a crucial goal in clinical medicine so that women have equal access and representation. Although approximately half (46%) of US neurology residents and fellows are female,^1^ proportions of female assistant, associate, and full professors are 49%, 41%, and 23%, respectively.^2^ This has far-reaching effects, from clinical publications to invited speakerships.^3,4^ Although a study noted increasing trends in female authorship in high-impact neurology journals,^5^ the current literature lacks evidence on a more informative benchmark—first and last authorship in randomized clinical trials (RCTs), which is typically considered for career advancement. This study assessed annual proportions and trends of female first and last authorship in neurovascular (stroke) RCTs from 2000 to 2021.

## Methods

In this cross-sectional study, we performed a PubMed search from January 1, 2000, to April 5, 2021, for the terms *neurovascular*, *vascular neurology*, *stroke neurovascular*, and *stroke vascular neurology*, with RCT or clinical trial as article types. The Thomas Jefferson University Institutional Review Board deemed the study exempt from approval and informed consent because it used publicly available data and was not considered human subjects research. The study followed the Strengthening the Reporting of Observational Studies in Epidemiology (STROBE) reporting guideline.

The gender of first and last authors was assessed based on the US Social Security Administration infant name database from 1932 to 2020. For names unidentified in this database and for non–English language articles (205 first authors and 168 last authors), author profiles were searched via a web-based search engine (Google) with the listed affiliation to determine gender. A χ^2^ test was performed comparing gender proportions. Male vs female authors were stratified yearly from 2000 to 2020; the year 2021 was not included. Simple linear regression tests were performed for trends for both genders. Subgroup analysis was done using publications from selected journals with a high impact factor (≥8): *Neurology*, *Stroke*, *Brain*, *The Lancet*, *The Lancet Neurology*, *JAMA Neurology*, *New England Journal of Medicine*, and *Annals of Neurology*. Statistical analyses were performed using Prism, 9th edition (GraphPad Software). Significance was set at *P* < .05 using 2-sided tests.

## Results

Our search yielded 1944 RCTs, of which 538 (27.7%) had female first authors and 289 (14.9%) had female last authors. High-impact journals yielded 477 (24.5%) of the RCTs, of which 125 (26.2%) had female first authors and 53 (11.1%) had female last authors (Table). Yearly proportions from 2000 to 2020 showed increasing trends for females having first and last authorship. There was no statistically significant trend in the high impact factor subgroup (Figure). In addition, a similar percentage of female and male first authors had unique names, defined as having only 1 publication (female: 412 [76.6%]; male: 1000 [71.1%]); however, 213 female last authors (73.7%) had unique names, and 983 male last authors (59.4%) did.

**Table.** Proportions of Males vs Females as First and Last Authors of Stroke Neurology Randomized Clinical Trials Published From 2000 to 2021

Authorship	Male, No. (%)	Female, No. (%)	*P* value^a^
**All journals (N = 1944)**			
First author	1406 (72.3)	538 (27.7)	<.001
Last author	1655 (85.1)	289 (14.9)	
**High-impact journals (N = 477)**			
First author	352 (73.8)	125 (26.2)	<.001
Last author	424 (88.9)	53 (11.1)	

^a^ χ^2^ test.

**Figure.**
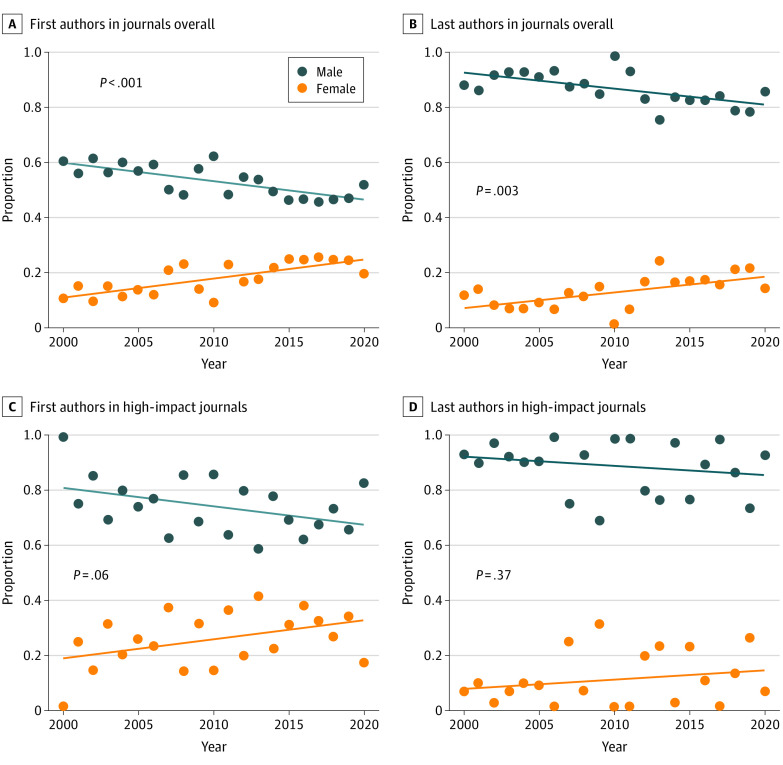
Trends in Gender of Authors of Stroke Neurology Randomized Clinical Trials Published From 2000 to 2020

## Discussion

In this cross-sectional study, we found that 27.7% of RCTs in academic stroke research from 2000 to 2021 had female first authors and 14.9% had female last authors. This difference may reflect a decreasing proportion of female neurologists as academic rank increases, because senior authors are traditionally listed last. Furthermore, there was an increasing trend for both first and last female authorship, which is similar to the findings of a cardiology study that showed increasing representation of women over time to approximately 30% of female first authors in cardiology RCTs in 2021.^6^ However, this trend did not hold in our subgroup of journals with a high impact factor. Although this result may be attributed to a lack of sufficient power owing to the small number of included articles that were published in high-impact journals (n = 477), the findings suggest that females may still experience challenges in being published as the first or last author in journals with a high impact factor. In addition, the lower percentage of unique last author names among men vs among women may indicate that a select few males authored multiple studies. The reduction in female authorship in 2020 may also reflect unique consequences of the COVID-19 pandemic as disproportionately affecting female researchers. A limitation of this study is that the results were based on gender associated with names in a binary fashion. Overall, although trends in academic research are beginning to reflect the increasing prevalence of female neurologists, there is still work to be done to promote gender parity in encouraging female authorship both as senior authors and in high-impact journals.
