# Error in the Figure

**DOI:** 10.1001/jamanetworkopen.2022.17084

**Published:** 2022-05-23

In the Research Letter titled “Gender Parity in Authorship of Published Randomized Clinical Trials in Stroke Neurology From 2000 to 2021,”^1^ published March 15, 2022, the data for male authors in the Figure, A, were plotted incorrectly. This article has been corrected.^1^
